# Higher Throughput Methods of Identifying T Cell Epitopes for Studying Outcomes of Altered Antigen Processing and Presentation

**DOI:** 10.3389/fimmu.2013.00430

**Published:** 2013-12-05

**Authors:** Evan W. Newell

**Affiliations:** ^1^Singapore Immunology Network, Agency for Science Technology and Research, Singapore

**Keywords:** epitopes, T-lymphocyte, antigen-specific T cell response, epitope mapping, MHC class I, MHC class II, peptide-MHC tetramers

## Abstract

Variation in the mechanisms that mediate antigen processing, MHC-loading, and presentation of peptides allows cells to significantly modulate the repertoire of peptides presented by both MHC class I or class II. To more quickly determine how these different modes or modulations of presentation translate into altered immune responses, higher throughput methods for identifying T cell epitopes are needed. Proteomics-based comprehensive cataloging of peptides eluted from MHC is a challenging but ideal way of identifying peptide sequences influenced by variable modes of processing and presentation. Several groups have already been successful with this approach and ongoing technical improvements will broaden its applicability. Subsequently, high content combinatorial peptide-MHC tetramer staining using mass cytometry, as we have recently described, should enable the broad assessment of how these changes are perceived by T cells and translated into an altered immune response. The importance of this analysis is highlighted by evidence that physiologically relevant variation in antigen processing and presentation as well as other factors can give rise to unpredictably different T cell responses.

## Variation in Antigen Processing and Presentation

Antigen processing and presentation is centrally important for T cell mediated adaptive immunity. Accordingly, the modes and mechanisms of peptide processing and presentation by MHC class I and class II molecules have been extensively studied and nicely described (1). Through these studies, numerous and diverse pathways have been identified for both MHC class I and class II. In both cases, the source of antigen can be exogenous, acquired through various endocytic pathways, or endogenous, synthesized, and processed by the antigen-presenting cell (APC) itself [reviewed in Ref. (2, 3)]. Far from simple, the molecular players involved in each of these pathways can also vary, allowing the APC to further modulate the repertoire of peptides being presented. For instance, in the case of MHC class I peptide loading, the subunit composition of the proteasome is an important factor. In addition to the constitutive proteasome, an altered subunit composition has been defined for both the immunoproteasome, induced by inflammatory signals (4), as well as the thymoproteasome, expressed by thymic cortical epithelial cells and specialized in providing peptides for T cell positive-selection (5). For MHC class II, in addition to variation in routes of processing, which include various forms of endocytosis and autophagocytosis (6), variable expression, and activities of lysosomal proteases such as the cathepsins can significantly modulate of the repertoire of epitopes presented by differing cell types under differing conditions (7). Peptide editing by HLA-DM, which appears to facilitate preferential loading of high-affinity MHC class II binding peptides, and the HLA-DM-inhibitory effects of HLA-DO on this process have also been well studied. Although exactly how HLA-DO influences the MHC class II binding peptide repertoire is still not completely clear, its role as an HLA-DM-competitor appears to be important for modulation of antigen presentation MHC class II in the thymus and in B lymphocytes (8–10). These examples represent only the tip of the iceberg in modulators of peptide processing and presentation for both MHC class I and class II. Diversity in antigen processing may be important for modulation of the resulting immune response as well providing alternatives for mechanisms evaded by the wide range of strategies employed by pathogens.

For a number of pathways alluded to above, including the example highlighting the importance of the thymoproteasome, several studies have demonstrated the consequences of distinct antigen processing and presentation pathways on the T cell response. Another dramatic example of alternate processing and its effects on the T cell mediated immune response was originally described by Unanue and colleagues for alternate processing pathways of the hen egg lysozyme (HEL) model antigen. In this system, some T cells react only with APCs loaded with free exogenous peptide (Type B antigens), while others, Type A antigen-specific T cells, can also react with antigen derived from endogenous processing of intact protein [reviewed in Ref. (11)]. This type of alternative antigen processing has now been shown to be involved in the generation of insulin-reactive T cells in diabetic mice (12, 13). Furthermore, in response to Influenza infection, evidence is emerging that several pathways can be used and that the classical pathways are not always the most important (2). Based on these and other examples, the importance of diversity and plasticity in antigen processing is clear and is also an attractive target for therapeutic immunomodulation. Going forward though, as modulating antigen processing continues to be investigated, more comprehensive means of directly measuring their influence on the repertoire of peptides presented as well as the epitope usage by T cells are needed.

## Toward Comprehensive Assessment of Epitopes and Their Usage by T Cells

One of the most profound conundrums of T cell immunology is the unpredictability of epitope use and what factors govern T cell epitope dominance (14). Although clearly not the only factor, one major contributor to the unpredictability of T cell epitope usage comes from the complexities in antigen processing and presentation, as described above. To address the relationship between antigen presentation and the antigen-specific T cell response, comprehensive methods for evaluating the identities and densities of peptides presented by APC have proven very useful. Much progress has been made since the first peptides were eluted from MHC, fractionated, and sequenced using Edman degradation (15, 16). Although difficult due to the very low abundance of any given peptide antigen species, much progress has come through the use of proteomic analysis of peptides eluted from MHC using mass spectrometry (17). With these methods, unbiased catalogs of endogenously processed and MHC class I or class II presented peptides can be obtained and number in the hundreds to thousands (17–19). However, caveats to this approach remain, including the very large number of cells required for analysis and a bias against low abundance and low affinity peptide ligands. In terms of sensitivity for rare peptide antigens and the requirements of very large numbers of input cells, progress can be expected from the rapidly advancing sensitivity of highly sophisticated mass spectrometers (20). Already though, this approach is being widely used. In particular, after early success in identifying melanoma tumor antigens (21), it is now playing a central role in an overall strategy for identification and validation of tumor associated T cell antigens (22). Advancements in methods of quantifying peptide abundances are allowing for accurately comparing repertoires of peptides presented by different cell types or changes induced by cytokine stimulation (23, 24). In conjunction with transcriptional profiling, it has also used to assess factors that contribute to changes in the repertoire of peptides presented by MHC. Through these studies, the repertoire of peptides presented by MHC is being found to be indicative of an integration of cellular processes influenced by the transcriptome, the metabolic status of the cell, and undoubtedly many other factors (25, 26). The concept of the repertoire of peptides presented by MHC as an integrative representation of cellular physiology is appealing and it is not surprising given the complexity and plasticity of antigen processing mechanisms. Another recent study used a targeted and quantitative approach to measure the levels of presentation of pre-identified peptides over the course of Vaccinia viral infection. This study found dramatic variation in peptide presentation levels over the course of infection – again indicative of the plasticity of antigen processing mechanisms. They also observed a strikingly unpredictable relationship between the levels of peptide being presented and the magnitude of the corresponding T cell response (27). Thus, through further application of proteomics-based assessment of antigen presentation, we can expect to learn a great deal about the effects of the various modes of processing and presentation. The index of antigens identified by these approaches can also be used for subsequent analysis of T cell responses.

**Figure 1:**
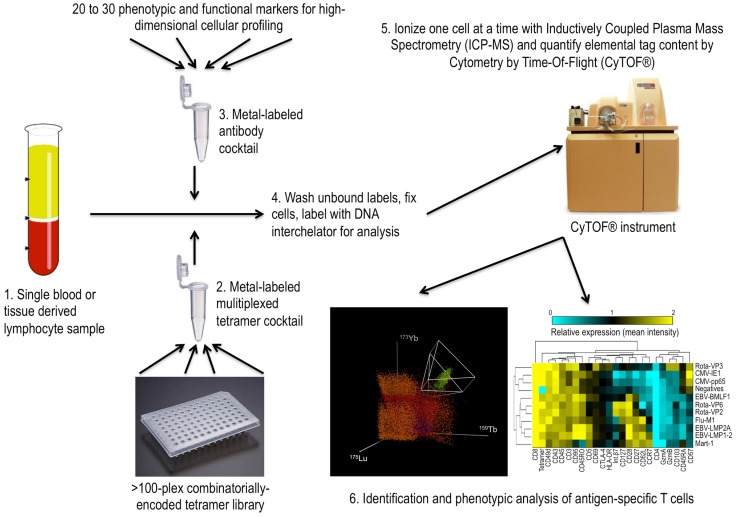
**Multiplex analysis of antigen-specific T cells with mass cytometry based combinatorial tetramer staining**. 1. Starting with a single blood or tissue sample, lymphocytes can be simultaneously stained with over 100 different combinatorially encoded heavy-metal labeled peptide-MHC tetramers. 2. To do this, tetramers are mixed into a single cocktail prior to cell staining. 3. The same cells are also stained with a cocktail of heavy-metal labeled antibodies for the purposes of probing the phenotypic and functional characteristics of the antigen-specific cells identified. 4. Prior to mass cytometry analysis, the cells are fixed, stained with metal-labled DNA interchelator, washed, and resuspended in water. 5. As they are introduced to the CyTOF^®^ mass cytometer, the cells are sprayed through a nebulizer and dried in argon before being ionized in plasma. Time-of-flight mass spectrometry is used to quantify each of the elemental tags on each cell. 6. Identities of antigen-specific cells are determined using multidimensional deconvolution algorithms and the phenotypic and functional characteristics of these cells can be compared through various analysis methods such as the clustergram shown.

In the past the only available approaches for tracking the antigen-specific T cell response were indirect and relied on response to antigen stimulation. More recently, the use of multimeric peptide-MHC complexes allow for direct detection of antigen-specific cells and accurate quantification regardless of T cell functional capacity (28). Despite some remaining limitations such as the relatively limited number of MHC alleles available for investigation and the dependence on relatively high-affinity T cell responses, recent progress has made the use of peptide-MHC multimers making their use more attractive for the broad analysis of antigen-specific T cell responses (29). In particular, the development of mass cytometry, a mass spectrometry based flow cytometry approach that uses heavy-metal tags instead of fluorophores (30), has greatly enhanced the utility of this approach. Although mass cytometry is still in its infancy and several limitations exist such as slower cellular throughput, reduced sensitivity, and an inability to sort or retain the cells being analyzed, the benefits of additional analysis parameters, especially when applied to multiplexing approaches are clear. Simultaneous assessment of many more characteristics of the antigen-specific cells identified with peptide-MHC multimers allows for a more comprehensive assessment of the phenotypic and functional characteristics of the antigen-specific T cells being identified (31). Also, through the use of multiple heavy-metal tags per peptide-MHC tetramer species as a part of a highly multiplexed assay, we recently demonstrated the assessment of more than 100 different T cell antigen-specificities in a single sample (see Figure 1) (32). In this study, the approach was used to screen for T cell reactivity of a large number of MHC class I binding peptides identified by binding prediction algorithms. The phenotypes of cells specific for each of the peptides were assessed in parallel and highlighted the remarkable influence of epitope usage on the phenotypes of the responding cells even for cells targeting different part of the same viral pathogens. For instance, in the case of EBV-specific cells consistent differences in phenotype were observed for cells targeting latency-associated proteins compared to lytic-cycle associated proteins. Similarly, we observed strikingly different phenotypes, trafficking patterns, and tissue residence for T cells specific for different parts of rotavirus in normal human donors.

Aside from previously characterized epitopes and predicted MHC-binding peptides, this highly multiplexed approach for identifying and characterizing antigen-specific T cells should also be useful for the screening of both MHC class I and class II ligands identified by proteomics for the purposes of investigating the consequences of variable antigen processing and presentation pathways discussed here. With room for improvement through dedication of additional parameters to multiplexed peptide-MHC tetramer analysis, already the number of candidate epitopes that can be screened is similar in scale to the number of peptides usually identified by mass spectrometry of MHC eluted peptides. Through improved sensitivity of proteomics-based methods of peptide identification and extension of the multiplexing capacity of the T cell analysis approach, it is hopeful that the goal of a truly comprehensive means of assessing epitope usage by antigen-specific T cells will be possible.

## Concluding Remarks

In line with the theme of this Frontiers in Immunology topic, the relationship between the complexities of antigen processing and presentation and the consequential antigen-specific T cell response were discussed. A great deal of successful research has led to understanding of the mechanisms, pathways, and molecular players of antigen processing and presentation, holding promise for powerful new ways of modulating the T cell mediated immune response. Nonetheless, due to the diversity of pathways and complexity of modulators involved, it is not yet simple to predict how perturbations will influence the repertoire of presented antigens or the resulting T cell response. Thus, the use of proteomics-based identification of peptide-MHC ligands as well as multiplexed methods for analyzing antigen-specific T cell responses should be useful for future studies on this topic.

## Conflict of Interest Statement

The author declares that the research was conducted in the absence of any commercial or financial relationships that could be construed as a potential conflict of interest.
